# Seasonal Influenza Prevention and Control Progress in Latin America and the Caribbean in the Context of the Global Influenza Strategy and the COVID-19 Pandemic

**DOI:** 10.4269/ajtmh.21-0339

**Published:** 2021-05-10

**Authors:** Andrea S. Vicari, Daniel Olson, Alba Vilajeliu, Jon K. Andrus, Alba Maria Ropero, David M. Morens, Ignacio José Santos, Eduardo Azziz-Baumgartner, Stephen Berman

**Affiliations:** 1 Health Emergencies Department, Pan American Health Organization, Washington, District of Columbia;; 2 Division of Pediatric Infectious Disease, University of Colorado School of Medicine, Aurora, Colorado;; 3 Department of Epidemiology, Colorado School of Public Health, Aurora, Colorado;; 4 Center for Global Health, Colorado School of Public Health, Aurora, Colorado;; 5 Comprehensive Family Immunization, Pan American Health Organization, Washington, District of Columbia;; 6 Department of Global Health, George Washington University Milken Institute of Public Health, Washington, District of Columbia;; 7 Division of Vaccines and Immunization, Center for Global Health, University of Colorado, Aurora, Colorado;; 8 Office of the Director, National Institute of Allergy and Infectious Diseases, National Institutes of Health, Bethesda, Maryland;; 9 Consejo de Salubridad General, Mexico City, Mexico;; 10 Influenza Division, U.S. Centers for Disease Control and Prevention, Atlanta, Georgia

## Abstract

Each year in Latin America and the Caribbean, seasonal influenza is associated with an estimated 36,500 respiratory deaths and 400,000 hospitalizations. Since the 2009 influenza A(H1N1) pandemic, the Region has made significant advances in the prevention and control of seasonal influenza, including improved surveillance systems, burden estimates, and vaccination of at-risk groups. The Global Influenza Strategy 2019–2030 provides a framework to strengthen these advances. Against the backdrop of this new framework, the University of Colorado convened in October 2020 its Immunization Advisory Group of Experts to review and discuss current surveillance, prevention, and control strategies for seasonal influenza in Latin America and the Caribbean, also in the context of the COVID-19 pandemic. This review identified five areas for action and made recommendations specific to each area. The Region should continue its efforts to strengthen surveillance and impact evaluations. Existing data on disease burden, seasonality patterns, and vaccination effectiveness should be used to inform decision-making at the country level as well as advocacy efforts for programmatic resources. Regional and country strategic plans should be prepared and include specific targets for 2030. Existing investments in influenza prevention and control, including for immunization programs, should be optimized. Finally, regional partnerships, such as the regional networks for syndromic surveillance and vaccine effectiveness evaluation (SARInet and REVELAC-i), should continue to play a critical role in continuous learning and standardization by sharing experiences and best practices among countries.

## INTRODUCTION

Influenza viruses are the archetypal human pathogens because of their recurrent seasonal epidemics and ever-looming pandemic threat. Globally, more than 650,000 people die of seasonal influenza-associated disease each year.^1^ In the Region of Americas, an estimated 51,674 seasonal influenza-associated respiratory deaths (95% CI: 41,007–71,710) and 772,000 hospitalizations (95% CI: 716,000–829,000) occur annually,^1–3^ of which approximately 36,500 respiratory deaths and over 400,000 hospitalizations occur specifically in Latin America and the Caribbean (LAC). In much of Latin America, respiratory disease mortality decreased gradually between 1998 and 2008,^4^ only to increase again following the 2009 influenza A(H1N1) pandemic.^5^ A systematic review and meta-analysis from 1980 to 2008 in LAC estimated an annual rate of 36,080 (95% CI: 28,550–43,610) influenza-like illnesses (ILIs) per 100,000 person-years.^6^ A systematic review of 98 studies that estimated globally or for specific world regions influenza-associated hospitalization rates for the period 2007–2018 found substantive variability in incidence estimates and underscored the need to strengthen the quality of such studies.^7^

Efforts to reduce this disease burden face substantial challenges, particularly in prioritizing activities and building capacities for prevention and control in advance of each influenza season. To that end, the WHO launched the Global Influenza Strategy 2019–2030 in March 2019,^8^ which provides countries and partners a framework for a comprehensive influenza disease approach to strengthen seasonal prevention and control as well as preparedness for future pandemics. The Global Influenza Strategy includes four strategic objectives: 1) promote research and innovation to address unmet public health needs; 2) strengthen global influenza surveillance, monitoring, and data utilization; 3) expand seasonal prevention and control policies and programs; and 4) strengthen influenza pandemic preparedness and response (Table 1).

**Table 1:** Strategic objectives and selected priority actions of the WHO Global Influenza Strategy 2019–2030^8^

Strategic objective*	Priority actions
1) Promote research and innovation to address unmet public health needs	1A: Promote research and innovation for improved and novel diagnostics, vaccines, and treatments against influenza
	1B: Promote operational research for influenza prevention, control, and program delivery
	1C: Promote research to better understand virus characteristics and host factors that drive the impact of influenza
2) Strengthen global influenza surveillance, monitoring, and data utilization	2A: Enhance, integrate, and expand virologic and disease surveillance
	2B: Build a strong evidence base for understanding the impact and burden of influenza
	2C: Develop effective influenza communication strategies across multiple sectors and between stakeholders
3) Expand seasonal influenza prevention and control policies and programs to protect the vulnerable	3A: Integrate nonpharmaceutical interventions into prevention and control programs
	3B: Design and implement evidence-based immunization policies and programs to reduce transmission and disease severity
	3C: Design and implement evidence-based treatment policies and programs to reduce morbidity and mortality
4) Strengthen pandemic preparedness and response for influenza to make the world safer	4A: Strengthen national, regional, and global planning to enable timely and effective pandemic readiness

* Strategic objectives 2 and 3 and priority actions 2A, 2B, and 3B are the focus of this article.

Influenza transmission in the last months of the 2019–2020 Northern hemisphere season and throughout the 2020 Southern hemisphere season has been historically low or even absent,^9^ including in countries with high testing frequency. This situation likely results from the stringent public health and social measures implemented in response to the COVID-19 pandemic. Whether similar lower transmission will repeat in the 2020–2021 Northern hemisphere and the 2021 Southern hemisphere seasons is uncertain and may depend on the continuation and adherence to nonpharmaceutical interventions. In this context, any discussion about seasonal influenza in LAC must include the evolving COVID-19 pandemic. Although the primary focus of this paper is prevention and control of seasonal influenza, when appropriate, the authors also note how principles of influenza prevention and control can be leveraged to prevent and mitigate COVID-19.

## UNIVERSITY OF COLORADO IMMUNIZATION ADVISORY GROUP OF EXPERTS MEETING AND OBJECTIVES

In October 2020, the University of Colorado Immunization Advisory Group of Experts (CU-IAGE) convened a group of international and regional experts to discuss current surveillance, prevention, and control strategies for seasonal influenza in LAC. The group included WHO, the Pan American Health Organization (PAHO, WHO Regional Office for the Americas), the CDC, the NIH, and experts from LAC countries. Participants were asked to submit high-impact papers before preceding and during the meeting. This paper summarizes CU-IAGE’s review of progress and challenges, and it provides recommendations (within the context of the Global Influenza Strategy 2019–2030) that LAC countries should consider to strengthen influenza surveillance and expand prevention and control strategies for seasonal influenza.

## STRENGTHENING INFLUENZA SURVEILLANCE, MONITORING, AND DATA UTILIZATION

### Progress and challenges of influenza surveillance (virologic and epidemiologic).

The Region of the Americas has gradually developed a comprehensive network of sentinel sites for syndromic surveillance of ILI sand severe acute respiratory infection (SARI) in ambulatory and hospitalized settings, respectively, generating weekly national surveillance reports. The PAHO has published surveillance reports detailing national surveillance data each week since August 2010.^10^ The collaboration among countries, PAHO, CDC, and the other partners was formalized in 2014, when the SARI Network for the Americas (SARInet) was established.^11^ SARInet provides a forum for professionals from Ministries of Health, reference laboratories, sentinel hospitals, and partner institutions to share experiences, collaborate, and define best practices to characterize and reduce the morbidity and mortality of influenza and other respiratory viruses in the Americas.

Important progress has resulted from these efforts to enhance, harmonize, and expand influenza disease and virologic surveillance. Currently, 25 and 17 of 35 PAHO Member States regularly report to PAHO/WHO their SARI and ILI surveillance data, respectively. An estimated 80% of the LAC population lives in countries with functional SARI/ILI surveillance. In the Americas, 29 National Influenza Centers—WHO-recognized laboratories within the Global Influenza Surveillance and Response System—and one WHO Collaborating Center at the CDC are integral components of such surveillance systems. A dozen countries use PAHOFlu, a regional web-based influenza and other respiratory virus surveillance data system. National surveillance information is shared each week globally with the Global Influenza Surveillance and Response System’s FluNet and FluID, global reporting systems for influenza virologic and epidemiologic data, respectively. Based on their surveillance data, a dozen countries use the Pandemic Influenza Severity Assessment methodology to evaluate in real time influenza transmission and severity and their joint impact on public health, health systems, and society.^12^ More effective prevention control efforts will require expanding national capacity for rapid risk assessment, effective information sharing, and integration with surveillance of zoonotic influenza cases. For example, predetermined thresholds and protocols based on data from prior years may be developed and implemented into (near) real-time influenza surveillance systems to alert on seasonal onset and manage expected disease burden.

Virologic surveillance is critical for influenza monitoring. For instance, virologic data demonstrate that both influenza B/Victoria and B/Yamagata circulate annually in all subregions,^13^ despite the dominance of influenza A infections.^14^ However, the Region of the Americas could benefit from more comprehensive data to characterize the medical and economic burden of each influenza lineage, especially influenza B.^13^

**Figure 1.**
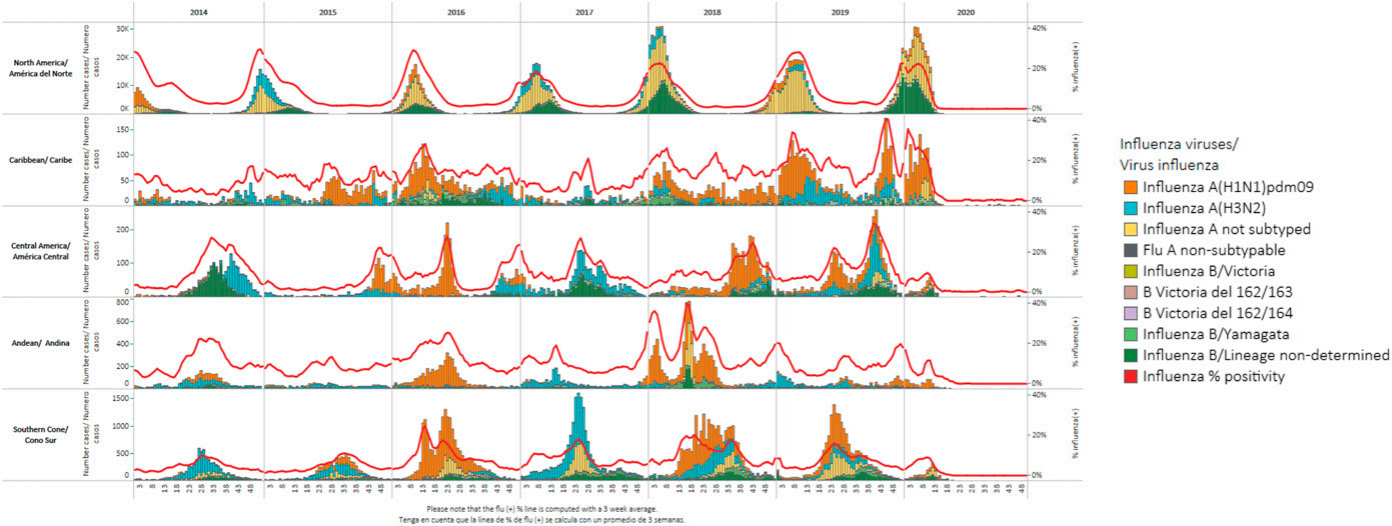
Influenza circulation by subregion in the Americas, January 2014–December 2020.^10^ Confirmed influenza cases, by week and subtype, and seasonal trends in five subregions of Latin America and the Caribbean, stratified by latitude. Greatest variation in seasonality is observed in Central America, the Caribbean, and Andes regions, with a more typical seasonal pattern observed in North and South America. All regions report a significant decrease in influenza cases in 2020. This figure appears in color at www.ajtmh.org.

Surveillance is also key to understanding seasonality, which in turn determines strains and timing for Northern and/or Southern hemisphere vaccines.^15^ Better understanding of seasonality would allow authorities to effectively anticipate the start of future seasons, mobilize communities with risk communication campaigns during epidemics (via hygiene and social distancing measures), and prioritize empiric antiviral treatment during peak epidemic activity. Defining influenza seasonal patterns is challenging in tropical areas, including in Central America and the Guyana shield.^16,17^ Significant heterogeneity exists between the timing of primary and secondary peaks across countries and subnational regions,^14^ though most countries in the LAC tropics have seasonal epidemics that start in May and peak between June and September (Figure 1).^18^ Although FluNet and national surveillance using various analytic approaches provide consistent national estimates of influenza timing, limited data exist at a subnational level.^19^

The gains resulting from PAHO’s efforts and SARInet’s have a spillover effect for surveillance of other respiratory pathogens. The regional response to COVID-19 is a prime example. In the new context of SARS-CoV-2 transmission, PAHO is working closely with countries to integrate COVID-19 into existing sentinel-based SARI/ILI surveillance. Previously, SARInet served as a platform to introduce confirmatory testing for other respiratory pathogens of public health importance, such as respiratory syncytial virus, in preparation for future novel monoclonal antibodies and vaccines; avian influenza viruses; and the Middle East Respiratory Syndrome coronavirus. With the emergence of SARS-CoV-2, PAHO leveraged the network to rapidly train personnel, distribute testing reagents, and validate national testing capacity. As a result, by the third week of February 2020, all countries in the Region of the Americas had established testing capacity at the national level or, for some countries and territories in the Caribbean, access to testing at a subregional laboratory.

### Progress and challenges in estimating disease burden.

Recent prospective studies demonstrate a significant medical burden of influenza in LACs. High-quality population-based cohort studies in Peru (2009–2015, all ages)^20^ and Nicaragua (2011–2013, infants)^21^ found laboratory-confirmed influenza incidence rates of 10.0 cases per 100 person-years (95% CI: 9.7–10.4) and 15.5 cases per 100 person-years (95% CI: 12.2–19.5) and found hospitalization rates of 0.7 (95% CI: 0.4–1.0) and 0.22 (95% CI: 0.03–1.55), respectively. Sentinel SARI surveillance systems in Chile (2012–2014) and Bolivia (2012–2017) also identified high rates of laboratory-confirmed influenza, hospitalization, and death.^22,23^ In the Peru cohort, less than half (44%; 95% CI: 35–54%) of participants with laboratory-confirmed influenza sought medical care.^20^ Additional high-quality data are needed on severe influenza, including respiratory and nonrespiratory (i.e., circulatory failure) causes of death, especially among high-risk groups.^1^ Opportunities to improve data quality include demographic data collection of case-patients and of persons tested for influenza (e.g., age), technological solutions for automatically accessioning information, and harnessing of electronic medical records in urban areas.

### Progress and challenges in estimating economic burden.

Ongoing studies are assessing the economic impact of influenza in LACs. A 2012–2013 study evaluating direct medical (outpatient consultation, medications, hospital fees), direct nonmedical (transportation, childcare), and indirect costs (lost wages) of hospitalized children with acute respiratory infections in El Salvador and Panama estimated a median societal cost of US$219 (interquartile range [IQR]: $101–416) and US$393 (IQR: $258–552) per case, respectively,^24^ with the government paying the majority of that cost. A 2008–2020 cohort study among children in Argentina found the median cost of hospitalization was US$529 (IQR: $362–789).^25^

A meta-analysis of studies from North America, Western Europe, Asia, and Australia found that 1.5–4.9 workdays are lost per laboratory-confirmed episode of influenza.^26^ Though data are limited, ILI likely also contributes significantly to work absenteeism in LAC.^6^ As percentage of the national gross domestic product, the estimated economic burden of seasonal influenza ranges from 2% to 5% in Brazil and from 4% to 8% in Argentina.^27^

These costs are likely underestimates because studies generally do not assess all direct and indirect costs related to illness, such as opportunity costs for workers and caregivers and reduced productivity while ill (“presenteeism”). The ability to perform economic analyses, especially in lower and middle-income countries (LMICs), is limited when economic burden evidence is unavailable.^1,15,28^

### CU-IAGE recommendations on surveillance and estimation of disease and economic burden.

The CU-IAGE recommended specific surveillance and burden estimation actions for countries to take. These include:

- Ensure strong surveillance systems to better characterize seasonality and virus circulation. Three areas could be prioritized in the medium term (i.e., next 3–5 years):
  - Reduce operational challenges faced by smaller and low-resource countries, such as maintaining supply chain, upkeep of laboratory equipment, and local data analysis and interpretation,
  - Improve processes for the timely shipment of a sufficient number of influenza-positive specimens to WHO Collaborating Centers responsible for genetic and antigenic characterization needed for the biannual vaccine composition meetings. This characterization is critical for global surveillance of influenza viruses with pandemic potential and for the biannual review of strains included in seasonal influenza vaccines.
  - Although a significant effort was devoted to improving the quality of surveillance data collection (e.g., influenza vaccine status among reported cases in countries contributing to vaccine effectiveness studies), surveillance data can be better used to mitigate and control seasonal influenza. For example, weekly surveillance data are used sporadically for national and subnational mobilization in favor of at-risk groups, health care providers, and the wider public, be it for antiviral treatment, influenza vaccination, or nonpharmacological interventions. In addition, few countries have protocols in place to detect unusual respiratory events that might signal a public health event of potential national or international concern.
- Generate accurate and comprehensive influenza medical burden estimates, particularly in severe disease and for the following high-risk groups:
  - pregnant women and health care personnel
  - persons with preexisting conditions
  - patients with extrapulmonary manifestations
- Standardize data collection and evaluation methods for economic studies in LMICs, including:
  - cost of illness in nonhospitalized patients
  - averted illness modeling, such as cost-effectiveness analyses of interventions and more accurate mathematical models with input from higher quality laboratory and epidemiologic data linked to mortality data
- Leverage evaluations done after influenza vaccine introduction and models of averted illnesses to tailor risk communication messages.

## EXPAND SEASONAL PREVENTION AND CONTROL POLICIES AND PROGRAMS, FOCUSING ON SEASONAL INFLUENZA IMMUNIZATION PROGRAMS

### Progress and challenges in seasonal influenza vaccination policy recommendations.

Influenza vaccination remains the best available measure to reduce the burden of severe influenza. In 2004, PAHO’s Technical Advisory Group (TAG) on Vaccine-Preventable Diseases recommended that all countries establish a seasonal influenza vaccination policy to vaccinate children 6–23 months of age, pregnant women, individuals with chronic illness, the elderly, and health workers. While acknowledging that surveillance is primarily for situational awareness and directing public health actions, the TAG specifically recommended that all countries strengthen surveillance systems to determine influenza disease burden, cost-effectiveness of influenza vaccine introduction, and optimal vaccination strategies, including formulation and timing.

**Figure 2.**
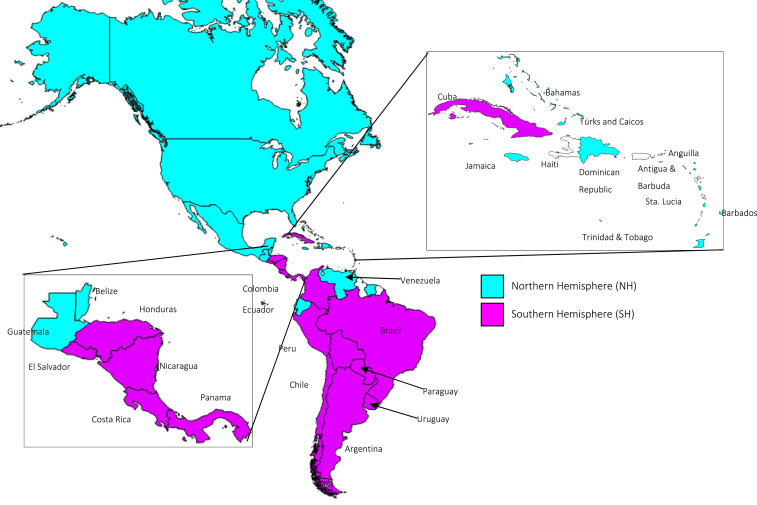
Distribution of countries in the Americas by influenza vaccine formulation used, 2020. Map showing distribution of Northern and Southern Hemisphere vaccine formulation use in 2020. Insets show the details in countries and territories of Central America and the Caribbean. Source: Country reports through the PAHO-WHO/UNICEF Joint Reporting Form 2020^31^ and PAHO Revolving Fund data for 2019–2020 Northern hemisphere and 2020 Southern hemisphere influenza seasons. This figure appears in color at www.ajtmh.org.

By 2004, 13 countries in the Region of the Americas had a policy for influenza vaccination.^29^ During the 2009 influenza A(H1N1) pandemic, the Region had one of the highest uptake of pandemic vaccines and vaccination rates; more than 145 million people were eventually immunized. Since then, seasonal influenza vaccination has continued to improve in the Americas.^30^ By 2019, 39 of the 51 (76.5%) countries and territories reported an influenza vaccination policy.^31^ Specifically, 39 countries recommend vaccinating health workers, 37 individuals with chronic diseases, 33 the elderly, 33 pregnant women, and 30 healthy children. In LAC, most countries start vaccinating in April with the southern hemisphere formulation, though there are exceptions (Figure 2).

In 2020, the WHO Strategic Advisory Group of Experts on Immunization (SAGE) recommended prioritizing risk groups for influenza vaccination during the COVID-19 pandemic, focusing on older adults and health workers.^32^ This would reduce influenza burden among groups who are also at high risk for severe COVID-19 disease, mitigate burden on health care systems, and protect health workers. The considerable overlap with population groups targeted for seasonal influenza vaccination offers the opportunity to leverage existing influenza vaccination programs to achieve high coverage for both vaccines.

### Progress and challenges of procurement and distribution of influenza vaccines.

Globally, the Americas has the highest distribution of influenza vaccine doses: 300 million annually or 271 per 1,000 population.^33^ Five countries in the Americas produce influenza vaccine or have fill/finish operations,^34^ and efforts are ongoing to develop capacity for regional vaccine production. Most LAC countries access and procure influenza vaccines through PAHO’s Revolving Fund, a mechanism that facilitates the joint purchase of vaccines (including influenza) at competitive prices for countries in the Americas.^35^ During the 2018–2019 season, approximately 25.4 million trivalent vaccine (TIV) doses and 2.4 million quadrivalent vaccine (QIV) doses were procured through this mechanism.^36^ Most LAC countries use Southern hemisphere standard-dose TIV, which is less expensive than standard-dose QIV ($1.2–3.4 versus $4.4–5.2 per dose, depending on pediatric/adult indication and presentation, for the 2020 Southern hemisphere formulation).^37^ The PAHO’s Revolving Fund also facilitates access to and distribution of vaccines for emergencies, as it is now the case for COVID-19 vaccines.

### Progress and challenges in seasonal influenza vaccination for pregnant women.

Influenza immunization programs expanded to include pregnant women following the 2012 WHO SAGE recommendations,^38^ given increased risk of severe maternal disease and mortality as well as increased risk of pregnancy loss, preterm birth, and low birthweight birth.^39,40^ Evidence indicates that maternal influenza immunization prevents influenza illnesses in pregnant women and their infants,^41–43^ although data about severe illness prevention in LMICs are limited.^44,45^

The PAHO and partners have conducted operational research studies in LAC to define optimal strategies to improve vaccination of pregnant women (and health workers).^46–49^ They have developed global and regional guidelines for national immunization program managers and policy makers to introduce influenza vaccination in pregnant women.^50,51^ The PAHO’s Regional Immunization Action Plan 2016–2020 includes an indicator to document maternal influenza immunization programs.^52^ This plan can reinforce the value of existing vaccination programs and the maternal and child health infrastructure.^51^

### Progress and challenges in monitoring seasonal influenza vaccination coverage.

Compared with other vaccines in the national immunization schedule, influenza vaccination faces unique technical and operational challenges: the requirements for annual vaccination, the need to define optimal vaccine formulation and vaccination timing in tropical areas, and the administration of two adequately spaced doses for naïve populations aged 6 months to 9 years. The 2015 TAG recommended vaccinating intensively prior to the peak of the influenza season, aiming for high vaccination coverage through a single campaign, followed by continued access through routine health services during the remainder of the season.^53^ Strategies to provide influenza vaccines year-round have also been discussed, such as alternating between Northern and Southern hemisphere formulations, extending the expiration date to permit extended use of a single-hemisphere formulation, and local vaccine manufacture with production timelines that align with local epidemiology.^54^

Other challenges include the need for tailored vaccination strategies and lower uptake and acceptance within certain high-risk groups, such as health workers.^55^ Although countries and territories in the Region of the Americas report influenza vaccine coverage by prioritized population groups annually (Table 2), accurate denominators to calculate coverage may be limited for certain high-risk groups, such as health workers and individuals with chronic diseases. Innovation is needed to monitor vaccination coverage more accurately and systematically. Social mobilization activities such as Vaccination Week of the Americas have supported massive influenza immunization campaigns; for example, Brazil successfully vaccinated over 59 million people during the Vaccination Week of 2019.^56^

**Table 2:** Seasonal influenza vaccine use in the Americas, 2019^68^

	Healthy children		Elderly adults		Other risk groups		
	Schedule	Coverage (%)*	Schedule	Coverage (%)	Health workers (%)	Pregnant women† (%)	Chronic diseases (%)¶
Anguilla	NA	NA	≥ 65 y	ND	Yes§	NA	Yes
Antigua and Barbuda	NA	NA	NA	NA	Yes	NA	Yes
Argentina	6 m–24 m	75	> 65 y	ND	100§	75	52
Aruba	< 2 y	ND	> 60 y	ND	Yes	Yes	Yes
Bahamas	> 6 m	ND	≥ 65 y	Yes	Yes	Yes	Yes
Barbados	NA	NA	NA	NA	Yes	NA	NA
Belize	6 m–35 m	83	≥ 65 y	36	46	72	Yes
Bermuda	6 m–18 y	17	> 65 y	12	1	10	2
Bonaire, St. Eustatius, and Saba	ND	ND	ND	ND	ND	ND	ND
Bolivia	6 m–23 m	80	> 60 y	75	100§	96	100§
Brazil	6 m–< 6 a	85	≥ 60 y	99	91	85	88
British Virgin Islands	NA	NA	NA	NA	2	NA	78
Canada	≥ 6 m	ND	≥ 65 y	70	Yes	Yes	Yes
Cayman Islands	≥ 6 m	ND	≥ 60 y	ND	Yes	Yes	Yes
Chile	6 m–5 y	74	≥ 65 y	68	97	94‖	94
Colombia	6 m–23 m	68	≥ 50 y	72	Yes	70	Yes
Costa Rica	6 m–< 6 a	ND	≥ 60 y	ND	Yes	Yes	Yes
Cuba	6 m–2 y	82	≥ 75 y	86	99	ND	85
Curacao	NA	NA	NA	NA	NA	NA	NA
Dominica	6 m–35 m	1	≥ 65 y	5	92	20	Yes
Dominican Republic	6 m–< 3 y	ND	Yes	ND	Yes	Yes	Yes
Ecuador	6 m–4 a	99	> 65 y	76	94	56	100§
El Salvador	6 m–11 m	57	≥ 60 y	42	100§	48	Yes
Grenada	6 m–35 m	ND	>6 5 y	ND	Yes	Yes	Yes
Guatemala	6 m–35 m	88	Yes‡	ND	Yes	Yes	Yes
Guyana	NA	NA	NA	NA	NA	NA	NA
Haiti	NA	NA	NA	NA	NA	NA	NA
Honduras	6 m–23 m†	57	> 60 y	68	85	85	100§
Jamaica	6 m–17 y	6	≥ 65 y	25	23	Yes‖	Yes
Mexico	6 m–59 m	91	≥ 60 y	94	100§	81	100
Montserrat	NA	NA	> 60 y	ND	Yes	NA	NA
Nicaragua	NA	NA	NA	NA	96	98‖	100§
Panama	6 m–59 m	71	> 60 y	83	95	63	Yes
Paraguay	6 m–36 m	32	≥ 60 y	30	Yes	Yes	Yes
Peru	< 2 y	58	> 60 y	47	Yes	37	Yes
Sint Maarten	NA	NA	NA	NA	NA	NA	NA
St. Kitts and Nevis	NA	NA	> 60 y	ND	Yes	7	7
St. Lucia	NA	NA	≥ 60 y	ND	Yes	Yes	Yes
St. Vincent	NA	NA	NA	NA	NA	NA	NA
Suriname	NA	NA	> 60 y	ND	Yes	Yes‖	Yes
Trinidad and Tobago	6 m–5 y	ND	> 65 y	ND	Yes	Yes‖	Yes
Turks and Caicos	6 m–3 y	ND	≥ 60 y	ND	Yes	NA	Yes
United States#	≥ 6 m–17 y	63	≥ 65 y	68	Yes	Yes	Yes
Uruguay	6 m–5 y	27	> 65 y	31	50	30	Yes
Venezuela	6 m–23 m	0	≥ 60 y	0	Yes	Yes	Yes

m = month(s); NA = not applicable; ND = no data reported; y = year(s); YES = influenza vaccination recommended (specific coverage not reported). Source: Country reports through the PAHO-WHO/UNICEF Joint Reporting Form (JRF), 2020; USA data source: https://www.cdc.gov/flu/fluvaxview/coverage-1819estimates.htm.

* Pediatric coverage formula: (2nd dose + single dose)/denominator × 100.

† With chronic disease.

‡ Institutionalized population.

§ Reported coverage data >100%.

‖ Pregnant (chronic condition or trimestral only).

¶ Countries including adults with chronic diseases in their policy.

# USA data for 2018–2019 influenza season.

### Progress and challenges in estimating influenza vaccine effectiveness and cost-effectiveness.

Reports of influenza vaccine effectiveness in LMIC countries are limited. In 2013, a network for influenza vaccine evaluations known as REVELAC-i for its acronym in Spanish (Red para la Evaluación de Vacunas en Latino América y el Caribe-influenza) was launched to better understand real-world vaccine effectiveness in LAC.^57^ As of 2019, 15 countries have joined the network. Data for 2013–2017 suggest that influenza vaccination programs in five South American countries prevented more than one-third of laboratory-confirmed influenza-associated hospitalizations in young children receiving the recommended two doses (vaccine effectiveness [VE]: 43%; 95% CI: 33–51%) and vaccinated older adults (VE: 41%; 95% CI: 28–52%).^58^ A comparison of 2019 influenza vaccine effectiveness in four Southern hemisphere countries—Australia, Chile, New Zealand, and South Africa—indicated VE estimates were also heterogeneous, with all-ages point estimates ranging from 7 to 70% for A(H1N1)pdm09, from 4% to 57% for A(H3N2), and from 29–66% for B.^59^ Systematic annual evaluations that leverage influenza surveillance data and national immunization program information can provide vaccine effectiveness estimates that ultimately quantify the number of hospitalizations and deaths averted through vaccination.

One global review found influenza vaccination to be cost-saving in children and mostly cost-effective in the elderly and pregnant women (< $50K/QALY).^60^ Randomized controlled trials of workplace-based vaccination programs in the United States have demonstrated improved clinical (febrile illness) and socioeconomic (work absenteeism, medical visit, medication usage) outcomes in years with a good match between vaccine and circulating strains.^61,62^ These socioeconomic outcomes informed the recommendation of annual influenza vaccine among all healthy adults in the United States.^63^ Such studies have not been replicated in LMICs.

Further efforts are needed to determine the potential impact and cost-effectiveness of QIV versus TIV in LAC as well as high-dose and/or adjuvanted vaccines for specific risk groups (e.g., older adults).^13^ Such information can inform health authorities because the ability to procure specific vaccines and target vaccination campaigns to key populations within a country depends on reliable disease burden data and evidence for benefits of vaccinating specific high-risk target groups.^15^ This information will also support effective messaging and communication critical for successful vaccine advocacy to targeted audiences and health workers.

### Future influenza vaccines.

Existing influenza vaccines are only effective when their formulation is well-matched to circulating strains. There is currently an international effort to develop influenza vaccines with broader and more long-lasting immunity (so-called “universal” influenza vaccines), independent of antigenic drift or hemagglutinin/neuraminidase subtype.^64,65^ Though still years away from clinical use, these vaccines may eventually allow less frequent administration and improve effectiveness and cost-effectiveness of influenza vaccination programs. In addition, cell-based influenza vaccines could shorten the interval between strain selection and vaccination, potentially improving vaccine effectiveness. Expanded use of adjuvanted influenza vaccines may also improve immunity in targeted populations. Vaccination against common causes of secondary bacterial pneumonia following influenza (*Haemophilus influenzae*, *Streptococcus pneumoniae*, *Staphylococcus aureus*, *Streptococcus pyogenes*) may decrease the risk of complications and hospitalizations associated with influenza and reduce secondary bacterial pneumonia.^64–66^

### CU-IAGE specific recommendations on strengthening seasonal influenza immunization programs.

The CU-IAGE recommended specific actions to strengthen seasonal influenza immunization programs at regional and national levels. These include:

- Strengthen regional mechanisms, including transfer of technologies agreements, to ensure regional production capacity to facilitate rapid access and distribution of seasonal influenza vaccine that could be leveraged in an event of a pandemic.
- Conduct knowledge, attitudes, and practices surveys to better understand attitudes and perceptions among specific groups and improve risk communications to increase vaccine uptake.
- Ensure that “lessons learned” are documented and follow-up on the recommendations from postintroduction influenza vaccine evaluations.
- For introduction of COVID-19 vaccines, leverage existing systems and explore synergies with influenza immunization programs for targeting similar high-risk groups.

## HIGH-LEVEL CU-IAGE RECOMMENDATIONS FOR STRENGTHENING SURVEILLANCE, PREVENTION, AND CONTROL OF SEASONAL INFLUENZA IN LATIN AMERICA AND THE CARIBBEAN

Focusing primarily on influenza, but in the context of the COVID-19 pandemic, the CU-IAGE issued the following high-level recommendations to guide the achievement of the 2030 goals of the Global Influenza Strategy in LAC:

- Define LAC regional plans to achieve seasonal influenza disease-specific targets for 2030 in alignment with goals of the Global Influenza Strategy 2019–2030 and the Immunization Agenda 2030.^67^
- Strengthen surveillance and disease and economic burden estimates (see section above).
- Optimize existing investments in influenza prevention and control, including strengthening influenza immunization programs (see section above) and the systematization of experience on supply chain and stockpiling of personal protection equipment, medical devices, drugs, and vaccines during the COVID-19 pandemic.
- Facilitate use of surveillance, seasonality, and vaccine effectiveness and impact data to inform country decisions and advocate for resources for influenza programs (including immunization programs).
- Leverage continuous learning through sharing experiences between countries through regional networks (i.e., SARInet and REVELAC-i).

The LAC countries and their partners should consider these recommendations in their planning for the upcoming influenza seasons. The progress toward the fulfillment of these recommendations should be integrated at the regional level in the ongoing activities for the monitoring and evaluation of the Global Influenza Strategy 2019–2030.
